# A novel approach of chemical mechanical polishing for cadmium zinc telluride wafers

**DOI:** 10.1038/srep26891

**Published:** 2016-05-26

**Authors:** Zhenyu Zhang, Bo Wang, Ping Zhou, Renke Kang, Bi Zhang, Dongming Guo

**Affiliations:** 1 Key Laboratory for Precision and Non-Traditional Machining Technology, Ministry of Education, Dalian University of Technology, Dalian 116024, China; 2 Changzhou Institute of Dalian University of Technology, Changzhou 213164, China; 3 State Key Laboratory of Metastable Materials Science and Technology, Yanshan University, Qinhuangdao 066004, China; 4 Department of Mechanical Engineering, University of Connecticut, Storrs, CT 06269, USA

## Abstract

A novel approach of chemical mechanical polishing (CMP) is developed for cadmium zinc telluride (CdZnTe or CZT) wafers. The approach uses environment-friendly slurry that consists of mainly silica, hydrogen peroxide, and citric acid. This is different from the previously reported slurries that are usually composed of strong acid, alkali, and bromine methanol, and are detrimental to the environment and operators. Surface roughness 0.5 nm and 4.7 nm are achieved for R_a_ and peak-to-valley (PV) values respectively in a measurement area of 70 × 50 μm^2^, using the developed novel approach. Fundamental polishing mechanisms are also investigated in terms of X-ray photoelectron spectroscopy (XPS) and electrochemical measurements. Hydrogen peroxide dominates the passivating process during the CMP of CZT wafers, indicating by the lowest passivation current density among silica, citric acid and hydrogen peroxide solution. Chemical reaction equations are proposed during CMP according to the XPS and electrochemical measurements.

Cadmium zinc telluride (CdZnTe or CZT) is a representative for the third-generation soft-brittle semiconductors in room temperature radiation detection, as well as a substrate for epitaxial growth of lattice-matched mercury cadmium telluride (HgCdTe or MCT) films used for infrared detectors^1^,^2^,^3^. Furthermore, CZT is widely used in medical imaging, homeland security, and spaceborne X-ray and gamma-ray astronomy^1^,^4^,^5^. This is attributed to its high gamma-ray absorption coefficient and high electrical resistivity, derived from the high atomic number and wide bandgap, respectively^1^. Nevertheless, CZT has soft and brittle nature^6^, which is different from the first and second-generation semiconductors with hard and brittle characteristics, such as silicon (Si)^7^ and gallium arsenide (GaAs) respectively, making it a hard-to-machine material. For instance, the hardness and fracture toughness of CZT are 1.21 GPa^8^ and 0.158 MPa.m^0.5^ respectively^9^, which are one twelfth (14.5 GPa)^10^ and one sixth (0.9–1.1 MPa m^0.5^)^11^ those of an Si crystal, correspondingly^12^.

Surface roughness has a significant effect on the electrical property and performance of CZT detectors, and therefore atomically smooth and defect-free surfaces are necessary to the high-performance CZT-based detectors^13^,^14^. Thus, the surface roughness root-mean-square (rms) <1 nm is required for a high performance CZT detector^14^,^15^. For this reason, surface processing techniques for CZT wafers have attracted attentions, and are investigated intensively^14^,^15^,^16^,^17^,^18^,^19^,^20^,^21^. Currently, lapping, mechanical polishing, chemical mechanical polishing (CMP), and chemical etching are usually employed to machine a CZT wafer. Firstly, free abrasives of alumina are widely used to lap and polish the surfaces of CZT wafers with different grain sizes in a sequence^16^,^17^. Nitric acid (HNO_3_) and bromine methanol (BM) are normally used in CMP^14^,^18^ and chemical etching^16^,^17^ respectively, to machine CZT wafers. Nonetheless, free abrasives of alumina are easy to embed in a CZT surface during lapping and polishing^18^. After embedding, the abrasives are difficult to remove in the successive processes. This results in the high surface roughness and low surface quality. Moreover, HNO_3_ is highly corrosive, and BM is toxic to the environment and operators. Hereby, it is necessary to develop a novel environment-friendly approach of CMP for CZT wafers to overcome the disadvantages of the conventional approaches.

Surface roughness is generally related to the measured area, i.e., smaller area leads to lower surface roughness. For example, surface roughness arithmetic average R_a_ and PV values of 0.32 and 3.29 nm are reported, respectively using atomic force microscopy (AFM) with a scanning area of 0.5 × 0.5 μm^2^ on a CZT surface^14^. With an increase in the scanning area to 5 × 5 μm^2^, R_a_ and PV values increase to 0.94 and 20.2 nm, correspondingly^14^. These CZT wafers are polished by HNO_3_ and BM^14^. Surface roughness rms of 0.74 nm is obtained on a CZT surface using AFM with a scanning area of 1 × 1 μm^2^ after CMP and chemical etching^15^. The rms value increases to 1.181 nm in a scanned surface of 20 × 20 μm^2^ which is mechanically polished by free abrasives of alumina, followed by chemical polishing of bromine, ethylene glycol, and sodium hydroxide (NaOH)^19^. Bromine and ethylene glycol are toxic, and NaOH is much corrosive. Surface roughness R_a_ reaches to 1.8 nm measured by AFM with a scanning area of 2 × 2 μm^2^, which is produced after mechanical polishing by free abrasives of alumina and diamond, followed by chemical etching of BM^20^. For decreased toxicity, iodine is dissolved in methanol replacing bromine in chemical etching of CZT wafers after mechanical polishing by diamond pastes, and R_a_ and PV values are 1.563 and 15.85 nm^21^, respectively with a measurement area of 180 × 130 μm^2^. However, both iodine and methanol are toxic, despite less toxicity of iodine than that of bromine. Surface roughness rms and PV values are 2.063 and 27.834 nm, respectively on the CZT surfaces after mechanical polishing by alumina abrasives^16^. Surface roughness increases to 3.855 and 95.762 nm, correspondingly after chemical etching using BM. As a result, BM deteriorates the surface roughness, rather than improving it. Undoubtedly, CMP and chemical etching play an important role to decreasing the surface roughness of the CZT wafers. It is a challenge to develop a novel environment-friendly approach to achieve the surface roughness <1 nm with a measurement area of 50 × 50 μm^2^.

In this study, a novel and yet environment-friendly CMP approach is developed for CZT wafers. The approach uses fixed abrasives of silicon carbide (SiC) in mechanical lapping, followed by CMP consisting of mainly silica (SiO_2_), hydrogen peroxide (H_2_O_2_), and citric acid. Finally, the polished surfaces of the CZT wafers are cleaned and dried using deionized water and compressed air, respectively. The approach demonstrates promising polishing results of surface roughness R_a_ and rms values <1 nm over a measurement area of 70 × 50 μm^2^.

Figure 1(a) shows the rough surface of the as-received CZT wafer after multi-wire sawing. Figure 1(b) shows an optical image of lapped surface on a CZT wafer. There were neither embedded grains nor cracks, except for micro-scratches. Figure 1(c) shows the polished surface after CMP using the proposed approach. The polished surface looked like a mirror, and was perfectly smooth, free of scratches and cracks. The surface roughness R_a_, rms, and PV values were 0.498, 0.626, 4.707 nm, respectively, with a measurement area of 70.6 × 53 μm^2^. Therefore, the object of surface roughness rms <1 nm is achieved using a novel approach with a measurement area of 50 × 50 μm^2^.

Figure 2 shows the XPS spectra of tellurium (Te) element on different CZT surfaces. All the three surfaces exhibit the Te^0^3d_5/2_, Te^4+^3d_5/2_, Te^0^3d_3/2_ and Te^4+^3d_3/2_ peaks on the as-received CZT wafers, citric acid and mixed slurry consisting of H_2_O_2_, SiO_2_ and citric acid, as shown in Fig. 3(a,c,d), respectively, except for Te^4+^3d_5/2_, Te^4+^3d_3/2_ and extremely weak Te^0^3d_5/2_ peaks on the CZT surface induced by H_2_O_2_, as seen in Fig. 3(b)^22^,^23^.

Figure 3 shows XPS spectra of the cadmium (Cd) element on various CZT wafers. The XPS spectra of the as-received CZT surface reveal Cd^0^3d_5/2_ and Cd^0^3d_3/2_ peaks, as illustrated in Fig. 3(a), which are different from the Cd^2+^3d_5/2_ and Cd^2+^3d_3/2_ peaks generated by H_2_O_2_, citric acid and mixed slurry of H_2_O_2_, SiO_2_ and citric acid, as observed in Fig. 3(b,c,d), correspondingly^19^,^24^.

Figure 4 shows the electrochemical curves of H_2_O_2_, SiO_2_, citric acid and mixed slurry made of H_2_O_2_, SiO_2_ and citric acid as a function of potential versus SCE. The corrosion potential is also referred to open circuit voltage (OCV). The corrosion potentials of SiO_2_, citric acid, mixed slurry and H_2_O_2_ are −0.31, −0.21, −0.2, +0.05 V, respectively, and their passivation current densities are 10^−6.5^, 10^−6.53^, 10^−6.77^, and 10^−6.96^ A cm^−2^, correspondingly, at the potential versus SCE of +0.4 V. At potential versus SCE of +1.2 V, the passivation current densities are 10^−6.45^, 10^−6.43^, 10^−6.17^, and 10^−6.11^ A cm^−2^, for H_2_O_2_, mixed slurry, citric acid, and SiO_2_, respectively^25^,^26^.

Fixed abrasives of SiC grains are used during lapping processes, which is effective in eliminating the embedding of free abrasives, as shown in Fig. 2(b). The fixed abrasive lapping is different from the previous findings, in which free abrasives are usually employed. Ultrafine SiC grains, such as mesh sizes of 2500, 5000, and 8000, are efficient in decreasing surface roughness, saving time and cost of CMP.

In the CMP slurry, H_2_O_2_ slowly decomposes into water and oxygen gas in air,


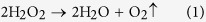


H_2_O_2_ solution is used as a medical disinfector. In this work, H_2_O_2_ solution is diluted by silica slurry and citric acid solution, and it is environment-friendly. Even the mixed slurry composed of H_2_O_2_, SiO_2_ and citric acid flushes hands, and the hands will turn into light yellow. After water flushing, the light yellow color fades. Silica slurry contains SiO_2_ and deionized water. SiO_2_ distributes widely in nature and occupies a weight percentage of 12% in the earth crust, such as stones mainly consisting of SiO_2_ and calcium carbonate (CaCO_3_). The size of SiO_2_ spheres played a significant effect on the material removal rate^27^, and therefore the diameters of SiO_2_ spheres varied from 25 to 118 nm, as shown in Fig. 5. Generally speaking, the distribution of particle size is important to controlling surface roughness, i.e. either a high material removal rate with large particles, or low surface roughness with relatively small particles. An appropriate distribution of particle size could obtain a balance between the material removal rate and surface roughness, resulting in a relatively high material removal rate and low surface roughness. Thus, the distribution of particle size in this study expects to produce coherent effect for the material removal rate and surface roughness, and achieve ultralow surface roughness at a relatively high material removal rate. Citric acid is a drink, and is popular in the food industry. During the final step of cleaning the CZT wafers, deionized water and compressed air are used to displacing the previously toxic etchants and cleaning agents, such as BM^20^, bromine-based etchants^14^, methanol^19^, and ethanol^18^. Both deionized water and compressed air are natural. Thus, the novel CMP approach for CZT wafers are environment-friendly, and can be applied to lapping, CMP and cleaning processes.

Fundamental polishing mechanisms are investigated using XPS and electrochemical measurements. In Fig. 4, the passivation current density of the H_2_O_2_ solution is the lowest among the four solutions, implying that the most compact passivation films can be formed on the CZT wafers. Citric acid is a pH adjustor in the CMP slurry, and its passivation effect is similar to that of the silica slurry. This is attributed to the similar curves of passivation current density between the citric acid and the silica slurry. In the mixed slurry, the corrosion potential decreases from +0.05 of H_2_O_2_ solution to −0.2 V, diluting by SiO_2_ slurry and citric acid. This leads to decreasing of the pH value of the H_2_O_2_ solution from 2.89 to 7.61 of the mixed slurry, whereas the passivation current density of the mixed slurry is the same as that of the H_2_O_2_ solution after potential versus SCE at +1.2 V. Consequently, H_2_O_2_ solution dominates the passivation current density, playing a key role in dissolving the CZT wafers and forming an ultrasmooth surface with the lowest surface roughness. Thereby, the chemical reaction equations of the H_2_O_2_ solution are proposed with CZT wafers^28^,^29^,^30^:


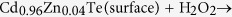



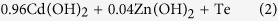



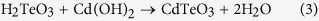



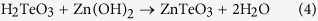



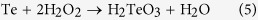



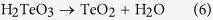


In Figs 2(a) and 3(a), Cd^0^3d, Te^0^3d, and Te^4+^3d are present. Cd^0^3d and Te^0^3d are derived from Cd_0.96_Zn_0.04_Te surface. Te^4+^3d valence state comes from the following chemical reaction:


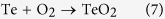


This is because of the as-received CZT wafers are exposed in air after multi-wire sawing from an ingot of CZT. Te usually enriches on the surfaces of the CZT wafers, and therefore Eq. (7) prevails. Accordingly, Cd is at the state of Cd^0^3d. With Eqs (2, 3, 4, 5, 6), the final reaction products are CdTeO_3_, ZnTeO_3_ and TeO_2_ under the function of the H_2_O_2_ solution. This is verified by the Te^4+^3d valence state in Fig. 2(b). As Cd(OH)_2_ and Zn(OH)_2_ are dissolved in H_2_O_2_ and mixed slurry with ion state, Eqs (3) and (4) have priority than Eq. (5) for an elementary substance. In the high concentration of the H_2_O_2_ solution, Eq. (5) reacts effectively, leading to an extremely small amount of Te left, as illustrated in Fig. 2(b). However, in the mixed slurry, the H_2_O_2_ solution is greatly diluted to a pH value of 7.61, Eq. (5) reacts ineffectively, resulting in a large amount Te appeared, as drawn in Fig. 2(d). With the effect of H_2_O_2_, Cd(OH)_2_ and CdTeO_3_ are produced, which are presented in Eqs (2) and (3), respectively. This is confirmed by Fig. 3(b,d) in the H_2_O_2_ and mixed slurry, correspondingly. Citric acid ionizes hydrogen (H) ions. The following equation happens:


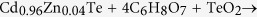



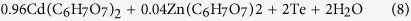


With Eq. (8), Cd^2+^3d is found in Fig. 3(c). Citric acid is a pH adjustor, passivation effect is similar to that of the silica slurry, as shown in Fig. 4. As a result, the dissolving effect of the citric acid for the CZT wafers is comparatively weak, resulting in both Te^4+^3d and Te^0^3d in Fig. 2(c). On the other hand, CZT crystals are easy to slip under stress even induced by high speed grinding, rather than forming amorphous phase, in terms of their low stacking fault energy (9.7 ± 1.7 mJ m^−2^)^6^,^9^. In consequence, the polished surfaces of CZT wafers are usually crystalline.

In summary, fixed abrasives of SiC are used in lapping CZT wafers to eliminate effectively the embedded free abrasives to save time and cost for subsequent CMP processes. A novel CMP approach is proposed in which the newly developed slurries consist of mainly H_2_O_2_, SiO_2_ and citric acid. The novel approach is environment-friendly. H_2_O_2_ solution dominates the passivation process, which is confirmed by electrochemical measurement. With the best passivation effect of H_2_O_2_ solution among four solutions, relatively strong Te^4+^3d peaks and an extremely small Te^0^3d_5/2_ peak are found.

## Methods

The as-received Cd_0.96_Zn_0.04_Te (111) wafers were 10 mm in length, 10 mm in width, and 1.5 mm in thickness, which were grown by the modified Bridgman method^1^. A precision polisher (YJ-Y380 of Shenyang Yanjia Co., Ltd. China) was employed to lap and polish the CZT wafers. SiC waterproof papers were put on a stainless steel plate as lapping pads. Four CZT wafers were fixed using a 502 glue on an aluminum plate of 150 mm in diameter uniformly along its periphery. CZT wafers were lapped using SiC papers with mesh sizes in a sequence of 2500, 5000, and 8000, and lapping time was set at 3, 2, 2 min, respectively. During lapping, the pressure of the lapping plate was 20 kPa, and rotation speeds of both the CZT wafers and SiC papers were 65 rpm. After lapping, the CZT wafers were cleaned using deionized wafer and dried by compressed air for further characterization by an optical microscope (Olympus).

After the optical characterization, the SiC papers were replaced by floss polishing pads on the stainless steel plate. The morphology and size of SiO_2_ spheres were measured by transmission electron microscopy (TEM, Tecnai spirit, FEI, Netherlands). The SiO_2_ spheres were used to produce silica slurry with a pH value ranging from 7 to 7.5, and a weight percentage of 60%. The oxidant was H_2_O_2_ solution that had a volume percentage of 40%. A volume ratio of 7 to 4 between the silica slurry and H_2_O_2_ solution was used to prepare the CMP slurry for the CZT wafers. Citric acid was used as a pH adjustor. The pH value of the CMP slurry varied from 4 to 4.5, which was decreased by the citric acid. During CMP, the rotation speeds of both the CZT wafers and floss polishing pads were 65 rpm. The polishing pressure and time were 30 kPa and 25 min, respectively. After CMP, the CZT wafers were cleaned and dried using deionized water and then compressed air.

Except the optical characterization, surface roughness and morphology of the CZT wafers were also measured by a precision non-contact surface profilometer (NewView 5022, Zygo, USA). X-ray photoelectron spectroscopy (XPS) was obtained by a VG ESCALAB MKII spectrometer with a magnesium Kα excitation source. Electrochemical measurement was performed on an advanced electrochemical system (PARSTAT 2273, Princeton Applied Research, Ametek, Inc.). The referenced and auxiliary electrodes were saturated calomel electrode (SCE) of potassium chloride (KCl) and platinum (Pt) with purity of 99.99%, respectively. In electrochemical measurement, the pH values of H_2_O_2_, citric acid, and mixed slurry consisting of H_2_O_2_, SO_2_, and citric acid were 2.89, 4.45, 7.61, respectively.

## Additional Information

**How to cite this article**: Zhang, Z. *et al.* A novel approach of chemical mechanical polishing for cadmium zinc telluride wafers. *Sci. Rep.* **6**, 26891; doi: 10.1038/srep26891 (2016).

## Figures and Tables

**Figure 1:**
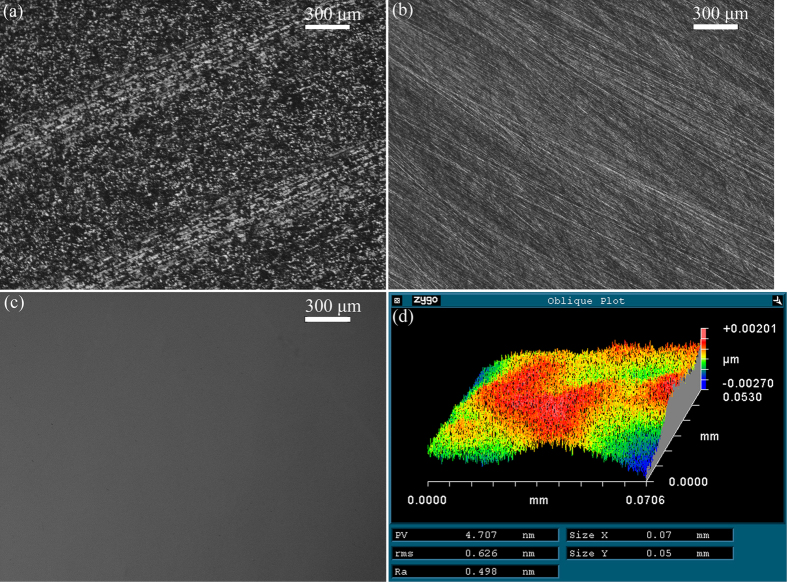
Optical images of (**a**) as-received, (**b**) lapped using SiC waterproof papers with mesh size of #2500, (**c**) polished by the developed approach of CMP CZT wafers, and (**d**) surface roughness and morphology measured by a surface profilometer (NewView 5022) after CMP.

**Figure 2:**
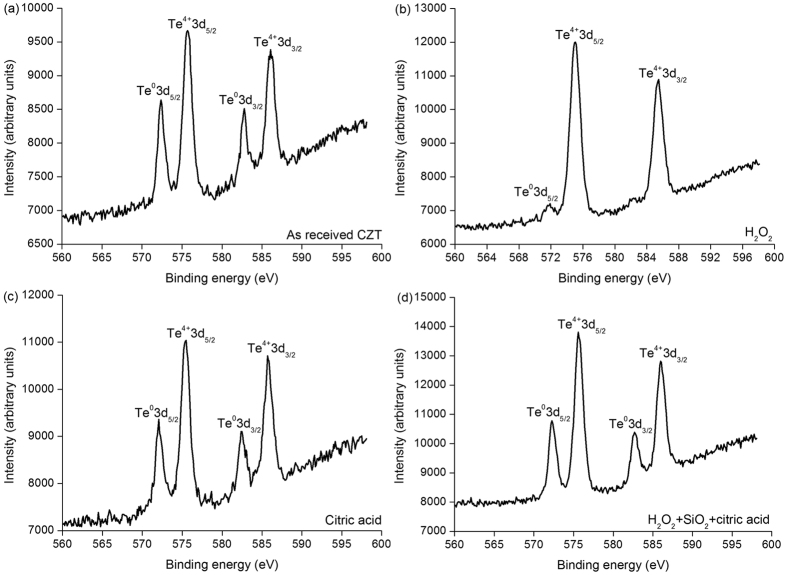
XPS spectra of Te element on CZT surfaces of (**a**) as-received, (**b**) H_2_O_2_, (**c**) citric acid and (**d**) mixed slurry consisting of H_2_O_2_, SiO_2_ and citric acid.

**Figure 3:**
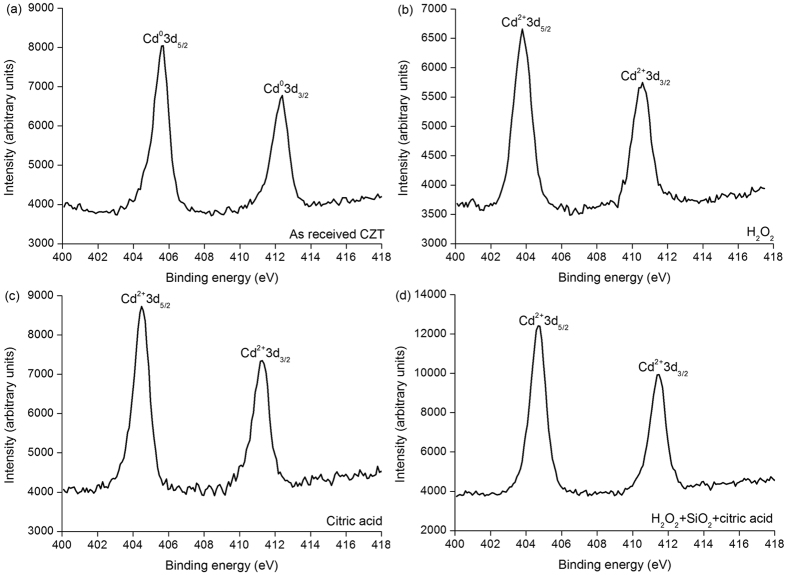
XPS spectra of Cd element on CZT surfaces of (**a**) as-received, (**b**) H_2_O_2_, (**c**) citric acid and (**d**) mixed slurry including H_2_O_2_, SiO_2_ and citric acid.

**Figure 4:**
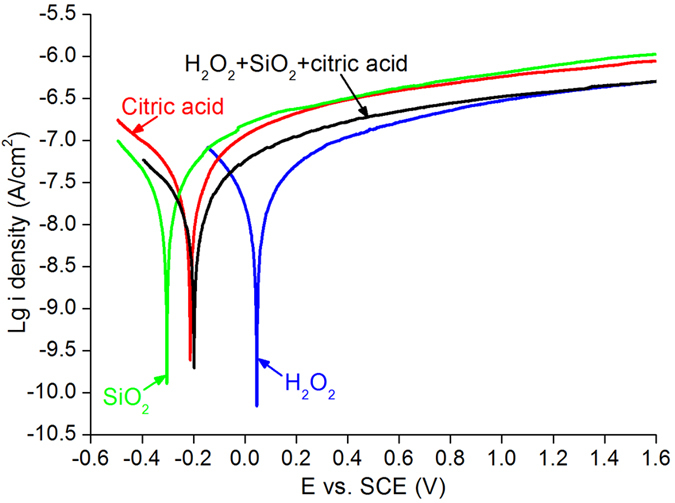
Electrochemical curves of H_2_O_2_, SiO_2_, citric acid and mixed slurry made of H_2_O_2_, SiO_2_ and citric acid as a function of potential versus SCE.

**Figure 5:**
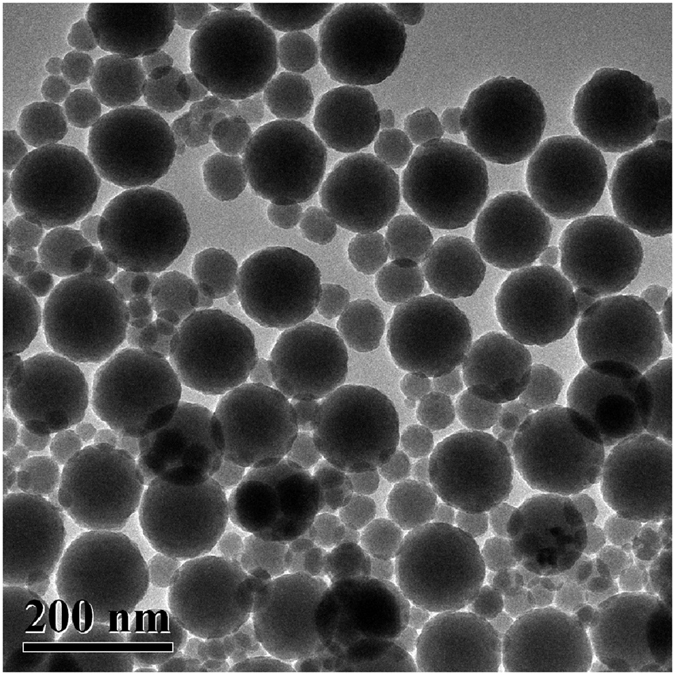
TEM image of silica spheres with diameters ranging from 25 to 118 nm.
